# Identification of volume overload hospitalizations among hemodialysis patients using administrative claims: a validation study

**DOI:** 10.1186/s12882-016-0384-6

**Published:** 2016-11-11

**Authors:** Magdalene M. Assimon, Thuy Nguyen, Suzanne L. Katsanos, Steven M. Brunelli, Jennifer E. Flythe

**Affiliations:** 1 Division of Nephrology and Hypertension, Department of Medicine, University of North Carolina Kidney Center, UNC School of Medicine, 7024 Burnett-Womack CB #7155, Chapel Hill, NC 27599-7155 USA; 2 Department of Epidemiology, UNC Gillings School of Global Public Health, Chapel Hill, NC USA; 3 Department of Medicine, UNC School of Medicine, Chapel Hill, NC USA; 4 DaVita Clinical Research, Minneapolis, MN USA; 5 Cecil G. Sheps Center for Health Services Research, University of North Carolina, Chapel Hill, NC USA

**Keywords:** Administrative claims, Hemodialysis, Hospitalization, ICD-9, Volume overload

## Abstract

**Background:**

High rates of volume overload hospitalizations may indicate inadequate dialysis facility fluid management. Administrative claims databases are often used to study such outcomes, but these data are generated for billing purposes and may not capture clinical nuance. It is unknown if volume overload admissions can be correctly identified in administrative data and if a single claims-based definition for volume overload can be used across epidemiologic surveillance studies, observational studies of exposure-outcome associations and quality assessments. We conducted a validation study to assess the accuracy of claims-based definitions for volume overload hospitalizations among hemodialysis patients.

**Methods:**

Data were taken from a random sample of 315 adult hemodialysis patients admitted to University of North Carolina Hospitals from January 2010 through June 2013. Standardized chart reviews were conducted to clinically adjudicate the presence or absence of volume overload at hospital admission. Claims-based definitions were constructed from varying combinations of fluid-related ICD-9 discharge diagnosis codes including fluid overload, pulmonary edema, pleural effusion, and heart failure. Using clinically adjudicated volume overload hospitalizations as the reference standard, validity metrics and their 95 % confidence intervals (CIs) were estimated for each definition.

**Results:**

Of the 315 hospital admissions, 77 (24.4 %) were clinically adjudicated as volume overload hospitalizations. The prevalence of claims-identified volume overload admissions varied across definitions, ranging from 1.6 to 37.1 %. When definitions were constructed with discharge diagnosis codes present in any billing position, volume overload hospitalizations defined by fluid overload, pleural effusion or heart failure diagnosis codes had the highest sensitivity, 81.8 % (95 % CI: 71.4 %, 89.7 %). Volume overload hospitalizations defined by pulmonary edema diagnosis codes had the highest specificity, 98.3 % (95 % CI: 95.8 %, 99.5 %). Definitions constructed with discharge diagnosis codes present in any billing position (versus the primary position) captured more false positive events.

**Conclusions:**

Prevalence and validity estimates of volume overload hospitalizations vary across claims-based definitions. A universal claims-based definition for volume overload hospitalizations may not apply to all clinical and research scenarios. Investigators and regulators need to consider the implications of misclassifying events when evaluating and monitoring hemodialysis patient volume overload admissions with administrative data. Claims-based definitions should be selected accordingly.

**Electronic supplementary material:**

The online version of this article (doi:10.1186/s12882-016-0384-6) contains supplementary material, which is available to authorized users.

## Background

The over 400,000 individuals receiving hemodialysis in the United States (U.S.) have exceedingly high rates of cardiovascular morbidity and mortality, with 30 % of hospitalizations and nearly 50 % of deaths attributed to cardiovascular causes [1]. Care of this complex population is expensive. In 2011, persons with end-stage kidney disease represented just 1.4 % of Medicare enrollees but consumed 6.3 % of the total Medicare budget [2]. Inadequate volume control is associated with both adverse cardiovascular outcomes and substantial healthcare costs among hemodialysis patients [3–5]. Volume-related hospital admissions are a significant driver of the cardiovascular hospitalization rate in the hemodialysis population, and estimated annual costs related to these encounters total over $250 million [1, 6].

Some volume overload hospitalizations may be preventable with better dialysis facility fluid management practices. For example, close attention to prescribed target (“estimated dry”) weight achievement at the end of each dialysis treatment as well as delivery of effective dietary salt and fluid restriction counseling by dialysis unit personnel may prevent some volume-related complications [4, 7, 8]. Tracking volume overload hospitalizations represents one potential strategy to measure and assess dialysis facility fluid management practices. The Medicare-based United States Renal Data System (USRDS), a national registry of end-stage kidney disease patients, is a readily available and cost effective data source often used to monitor and study cause-specific hospitalizations in the U.S. hemodialysis population.

Administrative claims data, such as that housed in the USRDS, are primarily generated for reimbursement and billing purposes. These data may not always capture clinical subtleties, potentially affecting the accuracy of claims-identified, cause-specific hospital admissions. For example, general population validation studies suggest that ~25 % of true heart failure hospitalizations are not captured by administrative claims data [9]. Prior evaluations of volume overload hospitalizations among hemodialysis patients were performed using USRDS data, each relying upon distinct combinations of discharge diagnosis and/or procedure codes to define events [6, 10, 11]. However, the validity of these claims-based definitions is unknown. In the medically complex hemodialysis population, restrictions on the number of diagnosis and procedure codes that can be billed per inpatient encounter, among other factors, may influence the ability of investigators to accurately identify cause-specific hospitalizations in administrative data. As such, when choosing claims-based volume overload definitions for observational studies, investigators must consider the implications of outcome misclassification and appropriately prioritize validity metrics (e.g. sensitivity and specificity) to optimize study accuracy. Study objectives and corresponding study design should guide claims-based outcome definition selection.

We undertook this study to evaluate the validity of several claims-based definitions for volume overload hospital admissions in the hemodialysis population using rigorous medical record reviews and medical center billing data.

## Methods

### Study population

This study included a random sample of 315 unique, adult maintenance hemodialysis patients admitted to University of North Carolina (UNC) Hospitals (Chapel Hill, NC) between January 1, 2010 and June 30, 2013. UNC Hospitals is a public academic medical center with over 800 inpatient beds and over 35,000 annual discharges. The study cohort consisted of hemodialysis patients who were: 1) ≥18 years of age and 2) admitted to medical or surgical services. We excluded patients who were: 1) receiving home hemodialysis or peritoneal dialysis or 2) newly designated as end-stage renal disease during the sampled admission. We selected 2010 as the study start year in order to exclude hospital admissions occurring before the Medicare policy change expanding the maximum number of billable discharge diagnoses per inpatient claim from 9 to 25 [12].

We performed a priori sample size calculations. Assuming a 20 % prevalence of volume overload admissions [1], a sample size of 298 patients would be needed to estimate a minimum specificity of 70 % with an acceptable lower 95 % confidence interval (CI) of at least 60 % [13]. This study was approved by the UNC Chapel Hill Institutional Review Board.

### Data sources

#### Overview

Data were obtained from the Carolina Data Warehouse for Health (CDW-H), a central data repository containing administrative healthcare data sourced from the UNC Health Care System. Detailed clinical data are not captured in this database and were abstracted from the electronic medical record by three clinicians (M.M.A., T.N. and S.L.K.).

#### Clinical adjudication of volume overload hospital admissions

For each sampled hospitalization, we conducted detailed clinical chart reviews to adjudicate the presence or absence of volume overload at the time of admission. We sought to identify hospitalizations of patients admitted *with* volume overload. Unlike other cardiovascular conditions, such as myocardial infarction, there is no established, objective definition for the clinical diagnosis of volume overload. Volume overloaded hemodialysis patients often present with a constellation of signs and symptoms indicative of fluid retention (e.g. shortness of breath, rales on lung auscultation, pulmonary edema on chest imaging, etc.) that may vary from individual to individual. Thus, an in-depth review of the medical record was necessary to capture all volume-related clinical findings associated with each sampled hospitalization. Medical chart notes (e.g. emergency department, admitting team and consult notes), chest and abdominal imaging reports, and cardiac procedure reports occurring within 24 h of admission were evaluated. Abstractors utilized a standardized data collection form (Additional file 1) to record symptoms, physical exam findings, imaging results, and clinical impressions. Each medical record was independently abstracted by two clinical reviewers who were blinded to the hospitalization’s billed diagnosis and procedure codes and to the abstraction results of the other reviewer. Inter-abstractor discrepancies in individual data elements were resolved by a board-certified nephrologist (J.E.F) Initial agreement between abstractors was high across all data elements, ranging 98.1 % (κ = 0.91) for subjective dyspnea to 100 % (κ = 1.00) for central venous pressure. After review and error resolution, consensus was reached on all abstracted charts.

**Fig. 1:**
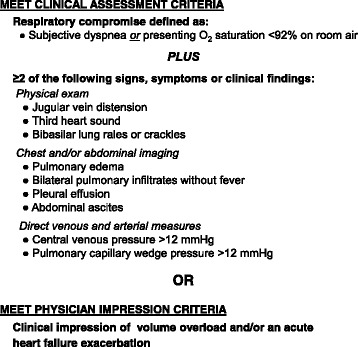
Reference standard criteria for adjudication of volume overload hospital admissions. Documented clinical assessment and physician impression criteria occurring within 24 h of admission were considered. Hospitalization events were adjudicated as volume overload admissions if either the clinical assessment criteria *or* physician assessment criteria for volume overload were met. Hospitalization events were adjudicated as non-volume overload admissions if they did not meet the physician impression criteria *and* did not meet the clinical criteria for volume overload

We created a standardized diagnostic algorithm to determine the presence or absence of volume overload at the time of admission based on the American College of Cardiology Foundation/American Heart Association and European Society of Cardiology guidelines [14, 15], and input from local nephrologists and cardiologists (Fig. 1). Diagnostic algorithm pre-testing revealed that a clinical criteria-based algorithm (e.g. symptoms, physical exam findings, and imaging results) failed to capture emergent presentations of volume overload requiring immediate treatment. In severe cases, imaging was not always performed prior to treatment (e.g. ultrafiltration). To capture such events, we expanded the algorithm to include clinical criteria *or* physician impression of volume overload. Furthermore, our diagnostic algorithm was developed to capture a range of volume overload severities. By design, our clinical definition does not distinguish patients admitted *for* the indication of volume overload from patients admitted *with* volume overload. Both scenarios were identified by our algorithm as clinically adjudicated volume overload events.

We applied the diagnostic algorithm to the abstracted data. Hospitalizations were adjudicated as volume overload admissions if either the clinical assessment criteria *or* the physician impression criteria for volume overload were met. Otherwise, hospitalizations were adjudicated as non-volume overload admissions. Agreement between volume overload admissions identified by clinical assessment criteria and admissions identified by physician impression criteria was high, 91.4 % (κ = 0.73).

#### Administrative claims-based definitions for volume overload hospital admissions

We obtained administrative data including demographics, billed hospital discharge diagnoses and procedure codes from the CDW-H for each sampled admission. We evaluated a range of administrative claims definitions for hospitalized volume overload. Definitions were constructed based upon literature precedent using various combinations of fluid overload, pulmonary edema, pleural effusion and heart failure International Classification of Diseases, Ninth Revision (ICD-9) discharge diagnosis codes (Table 1 and Additional file 2: Table S1) [6, 10, 11]. Primary validation analyses considered discharge diagnosis codes present in any position. Secondary analyses considered discharge diagnosis codes present in the: 1) primary billing position only and 2) primary or leading secondary billing position (separately). In additional secondary analyses, we evaluated the validity of claims-based volume overload definitions that included both fluid-related discharge diagnosis codes *and* the presence of a dialysis Current Procedural Terminology (CPT) procedure codes billed on the day of admission or the following day (Additional file 2: Table S2) [6]. The Medicare requirement of attending presence during in-hospital dialysis treatments for the billing of dialysis CPT codes may lead to inaccurate estimates of inpatient dialysis procedures in administrative data sources [16]. In our cohort, of the 59 clinically adjudicated cases who received dialysis within 24 h of admission (per medical chart documentation), only 39 (66.1 %) had a corresponding billed dialysis CPT procedure code. To avoid misclassification resulting from non-billed in-hospital dialysis treatments, validation analyses of claims-based definitions containing of discharge diagnosis codes were considered secondary, and results are presented in the supplemental material.

**Table 1:** Administrative claims-based definitions for volume overload hospital admissions

Definition number and description	Lead author (year of publication)^a^	ICD-9 discharge diagnosis codes^b^
1. Fluid overload^c^	Banerjee (2007) [10]	276.6, 276.69
2. Pulmonary edema	--	514, 518.4
3. Heart failure	--	398.91, 402.x1, 404.x1, 404.x3, 428^d^
4. Fluid overload^c^ or pulmonary edema	--	276.6, 276.69, 514, 518.4
5. Fluid overload^c^ or pleural effusion	Weinhandl (2015) [11]	276.6, 276.69, 511.9
6. Fluid overload^c^ or heart failure	--	276.6, 276.69, 398.91, 402.x1, 404.x1, 404.x3, 428^d^
7. Fluid overload^c^, pulmonary edema or heart failure	Arneson (2010) [6]	276.6, 276.69, 514, 518.4, 402.x1, 404.x1, 404.x3, 428^d^

*Abbreviations*: *ICD-9* International Classification of Diseases, Ninth Revision

^a^Denotes prior use of the ICD-9 diagnosis code combination to define volume overload hospitalizations

^b^Separate analyses evaluating definition validity considered ICD-9 diagnosis codes present in: 1) *any billing order position*, 2) *the primary billing position only* and 3) *the primary and leading secondary billing positions*

^c^Prior to October 1, 2010 the ICD-9 discharge diagnosis code 276.6 (fluid overload) was the only applicable code in existence. On October 1, 2010, ICD-9 diagnosis code 276.6 (fluid overload) became invalid and was replaced by more granular codes: 276.61 (transfusion associated circulatory overload) and 276.69 (other fluid overload). For hospitalizations with a discharge date prior to October 1, 2010 the ICD-9 code 276.6 was used to construct claims-based volume overload definitions. For hospitalizations with a discharge date on or after October 1, 2010 the ICD-9 code 276.69 was used to construct claims-based volume overload definitions

^d^Specified three digit ICD-9 diagnosis categories included all existing 4th and 5th digit diagnosis codes

### Statistical analyses

**Fig. 2:**
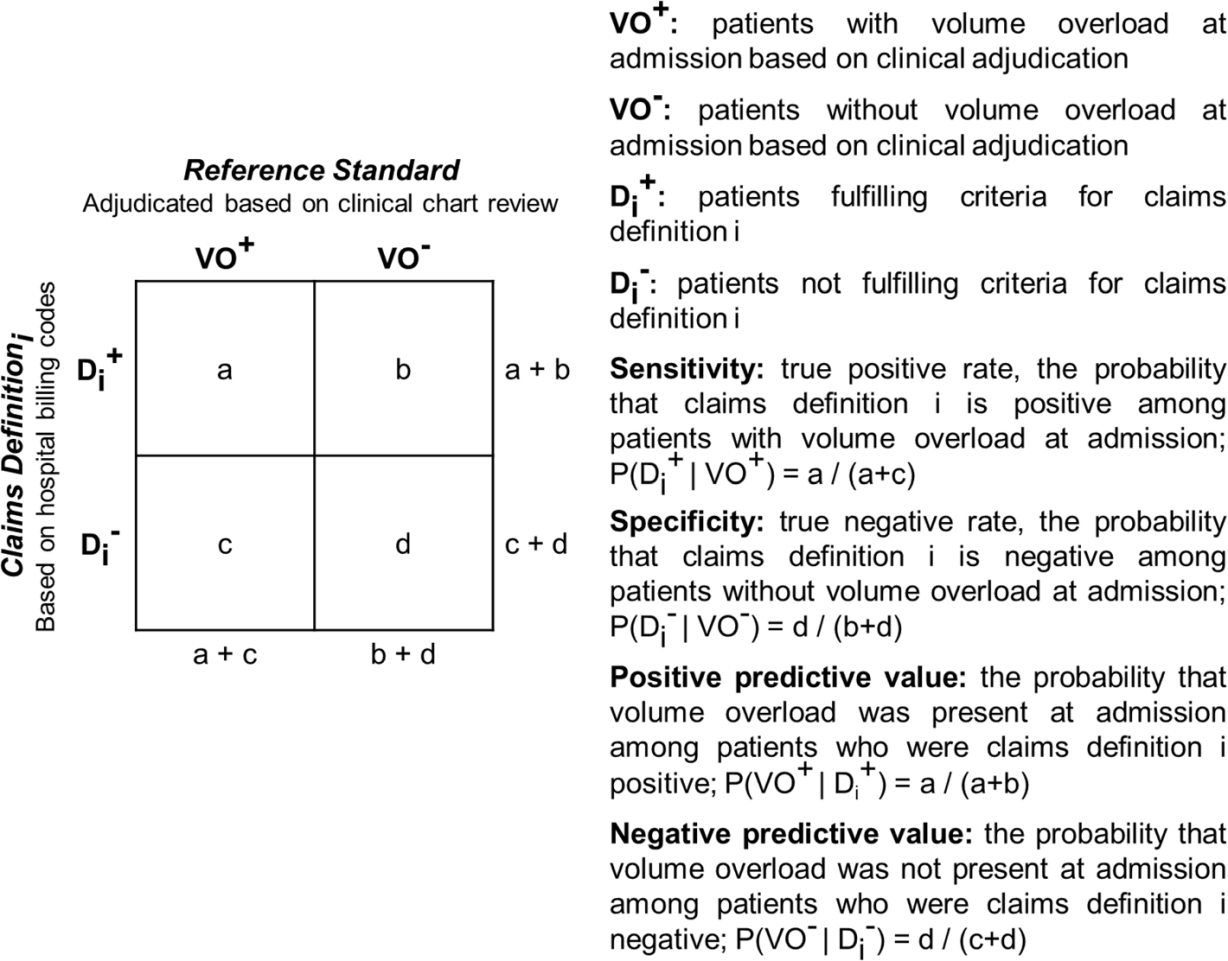
Computation and interpretation of validity measures

Analyses were performed using SAS version 9.3 (SAS Institute, Cary, NC). Data are presented as means and standard deviations or medians and interquartile ranges for continuous variables, and as frequencies and percentages for categorical variables. We computed the prevalence of clinically-adjudicated volume overload admissions and claims-identified volume overload admissions in the study cohort. Using clinically adjudicated volume overload hospitalization as the reference standard, we computed sensitivity, specificity, positive predictive value (PPV), and negative predictive value (NPV) and their exact binomial 95 % CIs for each claims-based volume overload definition (Fig. 2).

We conducted sensitivity analyses to assess the robustness of our findings. On October 1, 2010, the ICD-9 diagnosis code for fluid overload, 276.6, became invalid and was replaced by more granular codes: 276.61 (transfusion associated circulatory overload) and 276.69 (other fluid overload). Thus, we repeated validity assessments in a cohort restricted to patients admitted on or after October 1, 2010. Second, since most administrative claims analyses of U.S. hemodialysis patients are conducted in Medicare-based databases, we repeated validity assessments in a cohort restricted to patients with Medicare as their primary insurer.

## Results

### Cohort characteristics

Study cohort characteristics are displayed in Table 2. In the 315 patient cohort, the mean age was 57 ± 14 years, 172 (54.6 %) were male, 195 (61.9 %) were black, 119 (37.8 %) had diabetes and 143 (45.4 %) had heart failure. The study cohort was similar to the U.S hemodialysis population in terms of age and sex. Consistent with regional demographics, the study cohort had a higher proportion of black patients compared to a broader, national cohort [1]. The median (quartile 1 – quartile 3) length of admission was 4 (2–8) days. Of the 315 sampled admissions, 77 (24.4 %) were clinically adjudicated as volume overload hospitalizations. Compared to patients without volume overload at admission, patients with volume overload were more likely to have a history of hypertension, coronary artery disease and heart failure.

**Table 2:** Study cohort characteristics

Characteristics	All *N* = 315^a^	Volume overloaded at admission^b^ *n* = 77 (24.4 %)	Not volume overloaded at admission^b^ *n* = 238 (75.6 %)
Age (years)	57 ± 14	57 ± 16	57 ± 14
Female	143 (45.4)	37 (48.1)	106 (44.5)
Race			
Black	195 (61.9)	45 (58.4)	150 (63.0)
White	82 (26.0)	22 (28.6)	60 (25.2)
Other	38 (12.1)	10 (13.0)	28 (11.8)
Medicare as primary payer	266 (84.4)	62 (80.5)	204 (85.7)
History of diabetes^c^	119 (37.8)	30 (39.0)	89 (37.4)
History of hypertension^c^	217 (68.9)	64 (83.1)	153 (64.3)
History of arterial disease^c^	150 (47.6)	43 (55.8)	107 (45.0)
History of heart failure^c^	143 (45.4)	60 (77.9)	83 (34.9)
Length of hospital stay (days)^d^	4 (2–8)	3 (2–6)	4 (2–8)
Admitting service			
Medicine	257 (81.6)	74 (96.1)	183 (76.9)
Surgery	58 (18.4)	3 (3.9)	55 (23.1)
# of billed ICD-9 discharge diagnosis codes	14 (11–19)	16 (12–21)	13 (10–18)
Dialysis CPT procedure code billed on day of admission or the following day^e^	150 (48.5) [*N* = 309]	42 (56.0) [*n* = 75]	108 (46.2) [*n* = 234]
Recent TTE ejection fraction^f^			
< 35 %	4 (6.1)	3 (21.4)	1 (1.9)
35–54 %	30 (45.5)	4 (28.6)	27 (50.0)
≥ 55 %	32 (48.5) [*N* = 66]	7 (50.0) [*n* = 14]	25 (48.1) [*n* = 52]

Values presented as mean ± standard deviation, median (quartile 1 – quartile 3) or n (%)

*Abbreviations*: *CPT* Current Procedural Terminology, *ICD-9* International Classification of Diseases, Ninth Revision, *TTE* transthoracic echocardiogram

^a^Except where noted

^b^Volume overload status at hospital admission based on clinical adjudication

^c^Comorbid conditions were captured using all available administrative data occurring before the sampled hospitalization. ICD-9 codes for: diabetes included 250.xx; hypertension included 401.xx-405.xx (except 402.11), 402.91, 404.11, 404.13, 404.91, 404.93; arterial disease included 410.xx, 414.0x, 429.2x, 429.5x, 429.7x, 440.x, 440.2x, 440.3x, 440.8x, 440.9x, 443.9x; and heart failure included 398.91, 402.x1, 404.x1, 404.x3, 428.xx

^d^Length of hospital stay was computed as the discharge date minus the admission date. Patients admitted and discharged on the same day were assigned a length of stay = 0.5 days

^e^Six patients were admitted and discharged on the same day and were excluded from this computation because we were unable to determine if they received dialysis on the day following admission using inpatient administrative claims data. Dialysis CPT procedure codes used to identify inpatient dialysis treatments include: 90935, 90937, 90945 and 90947

^f^TTE conducted ≤1 year before sampled hospital admission

### Prevalence of volume overload admissions identified by administrative claims

**Fig. 3:**
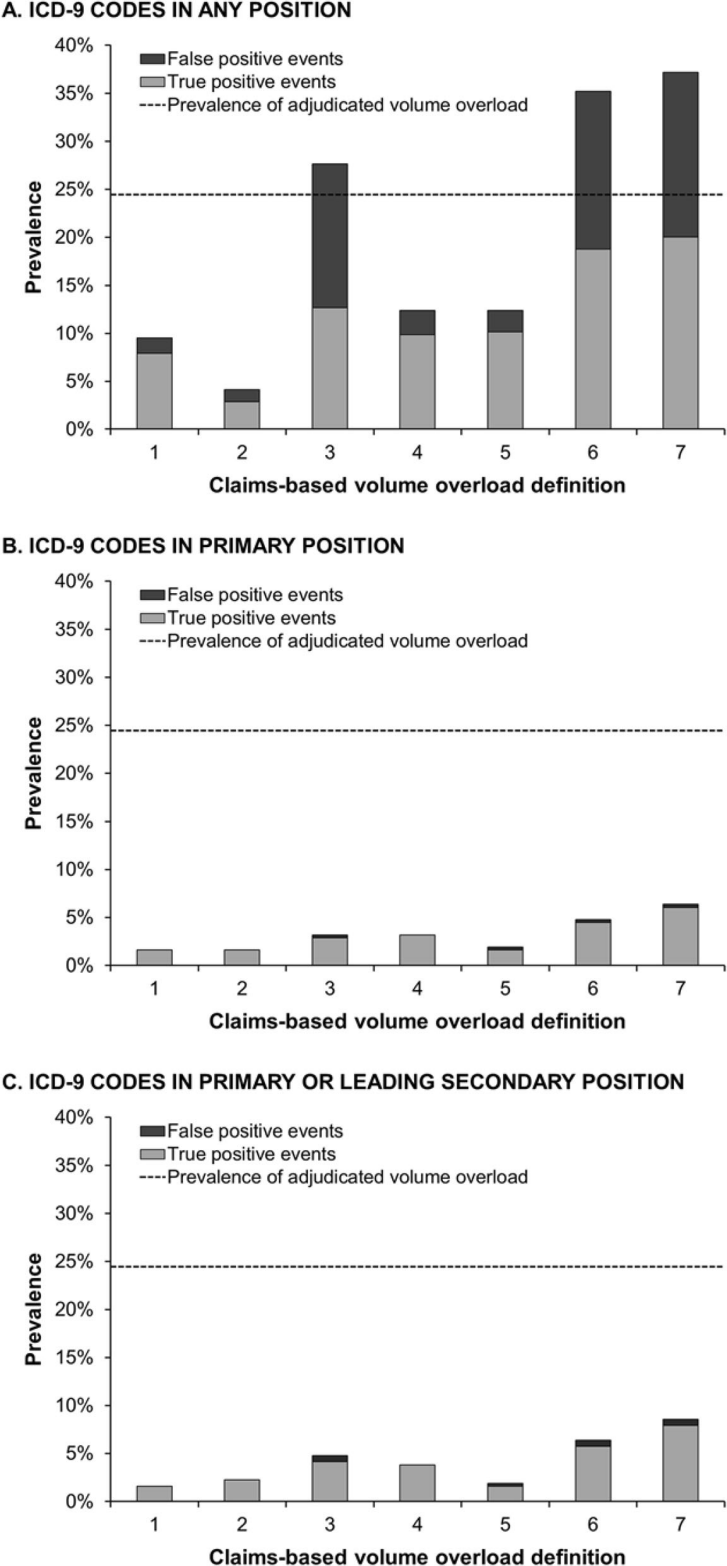
Prevalence of volume overload admissions identified by administrative claims definitions. The claims-based volume overload definition numbers on the x-axis correspond to the following definitions: 1) fluid overload; 2) pulmonary edema; 3) heart failure; 4) fluid overload or pulmonary edema; 5) fluid overload or pleural effusion, 6) fluid overload or heart failure; and 7) fluid overload, pulmonary edema or heart failure. The total bar height (*light gray portion* + *dark gray portion*) represents the prevalence of volume overload admissions in the study cohort identified using the specified claims-based definitions. Panel **a** depicts prevalence estimates when claims-based definitions were constructed considering ICD-9 discharge diagnosis codes in any position. Panel **b** depicts prevalence estimates when claims-based definitions were constructed considering discharge diagnosis codes in the primary position only. Panel **c** depicts prevalence estimates when claims-based definitions were constructed considering discharge diagnosis codes in the primary or leading secondary position. Abbreviations: ICD-9, International Classification of Diseases, Ninth Revision

The prevalence of volume overload hospitalizations differed across administrative claims-based definitions (Fig. 3). In primary analyses, when definitions were constructed considering diagnosis codes in any billing position, volume overload admission prevalence ranged from 4.1 % (definition 2, pulmonary edema) to 37.1 % (definition 7, fluid overload, pulmonary edema or heart failure) (Table 3). Definitions containing heart failure diagnosis codes (definitions 3, 6, and 7) overestimated volume overload admission prevalence, whereas definitions without heart failure codes (definitions 1, 2, 4, and 5) underestimated prevalence. Narrower definitions (i.e. those constructed with diagnosis codes billed in the primary position *or* in the primary or leading secondary positions) grossly underestimated volume overload admission prevalence. All claims-based definitions for volume overload hospitalizations comprised of discharge diagnosis *and* dialysis procedure codes underestimated volume overload prevalence (Additional file 2: Figure S1).

**Table 3:** Validity of administrative claims definitions for volume overload hospital admissions

Claims-based definition	n (%)^a^	SENS (95 % CI)^b^	SPEC (95 % CI)^b^	PPV (95 % CI)^b^	NPV (95 % CI)^b^
ICD-9 discharge codes could be *in any position*					
1. Fluid overload	30 (9.5)	32.5 (22.2, 44.1)	97.9 (95.2, 99.3)	83.3 (65.3, 94.4)	81.8 (76.8, 86.1)
2. Pulmonary edema	13 (4.1)	11.7 (5.5, 21.0)	98.3 (95.8, 99.5)	69.2 (38.6, 90.9)	77.5 (72.3, 82.1)
3. Heart failure	87 (27.6)	51.9 (40.3, 63.5)	80.3 (74.6, 85.1)	46.0 (35.2, 57.0)	83.8 (78.3, 88.3)
4. Fluid overload or pulmonary edema	39 (12.4)	40.3 (29.2, 52.1)	96.6 (93.5, 98.5)	79.5 (63.5, 90.7)	83.3 (78.4, 87.5)
5. Fluid overload or pleural effusion	39 (12.4)	41.6 (30.4, 53.4)	97.1 (94.0, 98.8)	82.1 (66.5, 92.5)	83.7 (78.8, 87.9)
6. Fluid overload or heart failure	111 (35.2)	76.6 (65.6, 85.5)	78.2 (72.4, 83.2)	53.2 (43.4, 62.7)	91.2 (86.4, 94.7)
7. Fluid overload, pulmonary edema or heart failure	117 (37.1)	81.8 (71.4, 89.7)	77.3 (71.5, 82.5)	53.8 (44.4, 63.1)	92.9 (88.4, 96.1)
ICD-9 discharge codes could be *in primary position only*					
1. Fluid overload	5 (1.6)	6.5 (2.1, 14.5)	100.0 (98.5, 100.0)	100.0 (47.8, 100.0)	76.8 (71.7, 81.4)
2. Pulmonary edema	5 (1.6)	6.5 (2.1, 14.5)	100.0 (98.5, 100.0)	100.0 (47.8, 100.0)	76.8 (71.7, 81.4)
3. Heart failure	10 (3.2)	11.7 (5.5, 21.0)	99.6 (97.7, 100.0)	90.0 (55.5, 99.7)	77.7 (72.6, 82.3)
4. Fluid overload or pulmonary edema	10 (3.2)	13.0 (6.4, 22.6)	100.0 (98.5, 100.0)	100.0 (69.2, 100.0)	78.0 (73.0, 82.6)
5. Fluid overload or pleural effusion	6 (1.9)	6.5 (2.1, 14.5)	99.6 (97.7, 100.0)	83.3 (35.9, 99.6)	76.7 (71.6, 81.3)
6. Fluid overload or heart failure	15 (4.8)	18.2 (10.3, 28.6)	99.6 (97.7, 100.0)	93.3 (68.1, 99.8)	79.0 (73.9, 83.5)
7. Fluid overload, pulmonary edema or heart failure	20 (6.3)	24.7 (15.6, 35.8)	99.6 (97.7, 100.0)	95.0 (75.1, 99.9)	80.3 (75.3, 84.7)
ICD-9 discharge codes could be *in primary or leading secondary positions*					
1. Fluid overload	5 (1.6)	6.5 (2.1, 14.5)	100.0 (98.5, 100.0)	100.0 (47.8, 100.0)	76.8 (71.7, 81.4)
2. Pulmonary edema	7 (2.2)	9.1 (3.7, 17.8)	100.0 (98.5, 100.0)	100.0 (59.0, 100.0)	77.3 (72.2, 81.8)
3. Heart failure	15 (4.8)	16.9 (9.3, 27.1)	99.2 (97.0, 99.9)	86.7 (59.5, 98.3)	78.7 (73.6, 83.2)
4. Fluid overload or pulmonary edema	12 (3.8)	15.6 (8.3, 25.6)	100.0 (98.5, 100.0)	100.0 (73.5, 100.0)	78.5 (73.5, 83.0)
5. Fluid overload or pleural effusion	6 (1.9)	6.5 (2.1, 14.5)	99.6 (97.7, 100.0)	83.3 (35.9, 99.6)	76.7 (71.6, 81.3)
6. Fluid overload or heart failure	20 (6.3)	23.4 (14.5, 34.4)	99.2 (97.0, 99.9)	90.0 (68.3, 98.8)	80.0 (75.0, 84.4)
7. Fluid overload, pulmonary edema or heart failure	27 (8.6)	32.5 (22.2, 44.1)	99.2 (97.0, 99.9)	92.6 (75.7, 99.1)	81.9 (77.0, 86.2)

*Abbreviations*: *95 % CI* 95 % confidence interval, *ICD-9* International Classification of Diseases, Ninth Revision, *NPV* negative predictive value, *PPV* positive predictive value, *SENS* sensitivity, *SPEC* specificity

^a^Count (prevalence) of volume overload admissions identified by each administrative claims definition in the study cohort (*N* = 315)

^b^Validity estimates and 95 % CIs are expressed as percentages. Clinically adjudicated volume overload events, as outlined in Fig. 1, served as the reference standard. In the study cohort there were 77 adjudicated volume overload admissions

### Validity of claims-based definitions for volume overload admissions

Table 3 displays the number of events, sensitivity, specificity, PPV and NPV for diagnosis code-based definitions for volume overload hospitalizations. In primary analyses considering definitions with diagnosis codes in any position, validity estimates varied across claims-based definitions. Sensitivity ranged from 11.7 % (definition 2, pulmonary edema) to 81.8 % (definition 7, fluid overload, pulmonary edema or heart failure). Specificity ranged from 77.3 % (definition 7 fluid overload, pulmonary edema or heart failure) to 98.3 % (definition 2, pulmonary edema). PPV ranged from 46.0 % (definition 3, heart failure) to 83.3 % (definition 1, fluid overload). NPV ranged from 77.5 % (definition 2, pulmonary edema) to 92.9 % (definition 7, fluid overload, pulmonary edema or heart failure). Compared to definitions considering ICD-9 codes in any position, definitions considering diagnosis codes in the primary position only, or in the primary or leading secondary positions had higher specificity and PPV and lower sensitivity and NPV.

Additional file 2: Table S3 displays validity estimates from secondary analyses considering claims-based volume definitions comprised of discharge diagnosis *and* dialysis procedure codes. In general, specificity and PPV were modestly higher, but sensitivity and NPV were commensurately lower when a dialysis procedure code was added to diagnosis code-based definitions. Validity results from cohorts restricted to patients admitted on or after October 1, 2010 (*n* = 287) and, separately, to patients with Medicare as the primary insurer (*n* = 266) were analogous to full cohort results (Additional file 2: Tables S4 and S5).

## Discussion

To our knowledge, this is the first study evaluating the accuracy of administrative claims definitions for volume overload hospitalizations in a hemodialysis population. Our study demonstrated that clinically adjudicated volume overload hospitalization prevalence differed from claims-derived prevalence estimates. In general, claims-based definitions had high specificity and low sensitivity. Our data suggest that certain claims-based definitions for volume overload hospitalizations could: 1) generate inaccurate estimates of temporal trends in disease surveillance programs; 2) misestimate the contribution of volume-related admissions to overall hemodialysis population health care utilization costs; or 3) render inaccurate estimates in observational studies seeking to understand how exposures impact rates of volume overload hospitalizations.

Existing data reveal that volume related-factors such as chronic volume expansion, interdialytic weight gain, and ultrafiltration rate contribute to the high hospitalization and mortality rates experienced by hemodialysis patients [3, 5, 17–20]. Thus, there is growing interest in identifying, quantifying and monitoring associated outcomes such as volume overload hospitalizations. To detect cause-specific hospitalizations, investigators and regulators typically rely on diagnosis and procedure codes in administrative healthcare databases such as the USRDS. However, administrative healthcare data may be inaccurate or incomplete for a variety of reasons. First, available diagnosis and procedure codes may not accurately identify the clinical condition of interest [21, 22]. Second, medical record documentation, coding and billing practices may vary across healthcare providers or institutions, creating data inconsistencies [23, 24]. Third, only a limited number of discharge diagnosis codes per hospitalization can be billed to insurers, possibly reducing clinical event ascertainment. Fourth, patients could receive treatment at a hospital or clinic without insurance filing, rendering administrative data sources incomplete [22, 23, 25]. While administrative databases are often the most accessible data sources, they may not be the most accurate. Potential data shortcomings must be considered when defining clinical outcomes.

In claims-based studies of hemodialysis patients, investigators have defined volume overload hospitalizations using a variety of fluid-related discharge diagnosis code combinations (e.g. fluid overload, pulmonary edema, pleural effusion, and heart failure) in varying billing positions (Additional file 2: Table S1) [6, 10, 11]. Banerjee et al. defined volume overload hospitalizations as the presence of a fluid overload or pulmonary edema discharge diagnosis code (separately) in any billing position [10]. Others have employed more restrictive definitions. Arneson and colleagues considered several fluid-related diagnosis codes (e.g. fluid overload, pulmonary edema, heart failure) present in the primary billing position only [6]. Whereas Weinhandl et al. defined volume overload hospital admissions as the presence of a fluid overload or pleural effusion discharge diagnosis code in the primary position only, or in the primary or leading secondary positions (separately) [11]. Not surprisingly, we found that broader (versus narrower) definitions identified more true positive volume overload admission events, but did so at the expense of capturing more false positive events. Most notably, we observed that claims-based definitions containing heart failure diagnosis codes (definitions 3, 6 and 7 with codes considered in any position) had the greatest tendency to identify false positive events. This finding may be attributable to the fact that some ICD-9 codes can be used to bill for both chronic stable heart failure and acute heart failure events.

Some investigators have identified volume overload admissions using discharge diagnosis codes in conjunction with dialysis procedure codes. For example, the claims-based definition used by Arneson et al. included fluid-related discharge diagnosis codes *and* also required the presence of a dialysis procedure code billed on the day of admission or the following day [6]. Inclusion of disease-specific procedure codes often increases definition specificity [23]. As anticipated, when we added dialysis procedure codes to diagnosis code-based definitions, we observed gains in specificity paired with reductions in sensitivity. However, the overall impact on validity estimates was minimal. This finding may, in part, be attributable to a hospital’s tendency to adhere to a patient’s outpatient hemodialysis schedule. Based solely on schedule, regardless of clinical presentation, greater than a third of all patients would be expected to receive dialysis within 24 to 36 h of admission. Furthermore, Medicare billing rules may impact the accuracy of claims-based definitions relying on dialysis procedure codes. Hospitals cannot bill dialysis CPT codes for treatments provided without the physical presence of the attending physician during the dialysis session [16]. In administrative data, this billing rule may lead to underestimation of dialysis procedures in academic environments where trainees supervise emergent overnight or weekend dialysis without in-hospital attending presence and in community hospitals where remote nephrology coverage is common. Thus, to maximize definition stability across clinical practice environments and to avoid outcome misclassification related to billing rules, it may be prudent to omit dialysis procedure CPT codes from claims-based definitions for volume overload hospital admissions.

Dialysis patient clinical complexity may also impact accuracy of claims-defined, cause-specific hospitalizations. A limited number of diagnosis codes can be billed for each hospital encounter. Most often, payers reimburse hospitals for inpatient services based upon billed Medicare Severity Diagnosis Related Groups (MS-DRGs). The patient’s primary (or principal) discharge diagnosis in combination with other factors such as patient sex, discharge status, complications and/or comorbidities documented as secondary discharge diagnoses, medical procedures performed, and length of stay, determine the assigned MS-DRG and corresponding level of reimbursement. Hemodialysis patients often have multiple comorbidities and are treated for numerous clinical conditions during hospitalizations, resulting in a wide range of potential discharge diagnoses from which to choose for coding and billing purposes. Medicare policies allow hospitals to preferentially select discharge diagnosis codes to maximize payment as long as they are supported by adequate medical record documentation [26]. The tendency of heart failure-based definitions to identify false positive volume overload admissions may be explained, in part, by a health system’s preference for coding more resource intensive conditions or comorbidities such as heart failure. Such practices likely vary across healthcare and reimbursement settings.

The ideal claims-based definition for volume overload hospital admissions would have perfect sensitivity (i.e. it would not capture any false negative events) and perfect specificity (i.e. it would not capture any false positive events). As claims-based definitions for clinical event identification are often imperfect, investigators must weigh the advantages and disadvantages of employing more sensitive versus more specific outcome definitions. Study objectives should drive this decision. More *sensitive* outcome definitions may be preferred in scenarios where enhanced inclusiveness is desired (e.g. epidemiologic surveillance studies) and when generalizability is important (e.g. quality assessment initiatives) [27, 28]. We demonstrated that claims-based volume overload admission definitions with poor sensitivity (<50.0 %) led to systematic underestimation of the volume-related hospital admission burden. This finding suggests that existing national prevalence estimates and temporal trends of volume-related hospitalizations may be conservative. On the other hand, more *specific* outcome definitions may be favored in observational studies examining exposure–outcome associations via relative effect measures. In the setting of non-differential outcome misclassification, implementation of claims-based volume overload admission outcome definitions with perfect specificity will generate unbiased risk ratio estimates [29]. Consideration of definition PPV and NPV is also important. Positive predictive value, like specificity, is an indicator of false positive event ascertainment, whereas NPV, like sensitivity, is an indicator of false negative event capture. However, unlike specificity and sensitivity, generalizability of PPV and NPV to a population other than the validation cohort depends on the prevalence of the outcome of interest in that population.

Strengths of our study include random selection of hospital admissions enabling estimation of the full spectrum of validity metrics, rigorous data abstraction by two independent reviewers, and utilization of standardized procedures for volume overload admission adjudication. Our study also has limitations. First, we used data from a single academic medical center. Validity estimates may not generalize to administrative data from hospitals with different billing and coding practices. Reassuringly, our study had a similar frequency of billed volume-related discharge diagnosis codes to prior investigations using USRDS data [6, 10, 11]. Second, an established, universal definition for the clinical diagnosis of volume overload does not exist. To address this limitation, we developed a standardized algorithm for clinical adjudication based on guideline body-accepted clinical and radiologic evidence of volume overload [14, 15]. Third, we investigated inpatient volume overload hospital admissions. Our validity estimates may not generalize to other hospital-based encounters such as observation stays or emergency department visits. Given that reimbursement rules and billing mechanisms differ across hospital encounter type, optimal volume overload definitions may vary across inpatient admissions, observation stays and emergency department visits [30, 31]. Future studies should assess the validity of claims-based definitions for volume-related observation and emergency department visits. Fourth, we evaluated inpatient admissions from January 2010 through June 2013. Our validity estimates may not generalize to periods outside of the study timeframe. Our modest sample size prevented evaluation of potential temporal coding trends on claims-based definition validity during the study period. Finally, we studied in-center hemodialysis patients. Results should not be extrapolated to excluded populations such as peritoneal dialysis or home hemodialysis patients or those with non-dialysis dependent chronic kidney disease.

## Conclusions

In conclusion, we investigated the validity of administrative claims-based definitions for volume overload hospital admissions in a cohort of maintenance hemodialysis patients. While administrative claims databases are efficient and cost-effective data sources, investigators and regulators must consider the implications of misclassifying volume overload admissions when studying, evaluating and monitoring such events. Our results suggest that a single, universal claims-based definition for volume overload hospitalizations may not be appropriate for all clinical and research scenarios.

## Additional files

Additional file 1: Chart abstraction form. Description of data: Standardized data collection form utilized by abstractors to record volume-related symptoms, physical exam findings, imaging results and physician clinical impressions. (PDF 103 kb)
Additional file 2: Supplemental tables and figures. Description of data: Includes the following supplemental tables and figures: **Table S1.** Detailed description of claims-based definitions for volume-related hospitalizations used in prior studies. **Table S2.** Administrative claims-based definitions for volume overload hospital admissions constructed with ICD-9 discharge diagnosis codes and a dialysis procedure codes. **Table S3.** Validity of administrative claims definitions for volume overload hospital admissions with an additional dialysis procedure requirement. **Table S4.** Validity of administrative claims definitions for volume overload hospital admissions among hemodialysis patients admitted on or after October 1, 2010. **Table S5.** Validity of administrative claims definitions for volume overload hospital admissions among hemodialysis patients with Medicare insurance. **Figure S1.** Prevalence of volume overload admissions identified by administrative claims definitions with the additional dialysis procedure requirement. (PDF 462 kb)

## Abbreviations

CDW-H: Carolina Data Warehouse for Health
CI: Confidence interval
CPT: Current Procedural Terminology
ICD-9: International Classification of Diseases, Ninth Revision
MS-DRG: Medicare Severity Diagnosis Related Group
NPV: Negative predictive value
PPV: Positive predictive value
SENS: Sensitivity
SPEC: Specificity
U.S.: United States
UNC: University of North Carolina
USRDS: United States Renal Data System
