# Supplementary material for: Identification of volume overload hospitalizations among hemodialysis patients using administrative claims: a validation study
Source: BMC Nephrol. 2016 Nov 11;17:173. doi: 10.1186/s12882-016-0384-6 (PMC5105303; doi:10.1186/s12882-016-0384-6)
Supplement: Additional file 1: — Chart abstraction form. Description of data: Standardized data collection form utilized by abstractors to record volume-related symptoms, physical exam findings, imaging results and physician clinical impressions. (PDF 103 kb) [file 12882_2016_384_MOESM1_ESM.pdf]

**Patient_SK**	
**Admission Date**	
**Abstracter Name**	
**Abstraction Date**	

Notes

Respiratory Compromise

Symptom/ Finding	Y	N	Qualitative	Date and Time	Chart location/ source
**Subjective dyspnea**					
**Instructions**			List how dyspnea was described. Examples: “patient presented with shortness of breath”; “patient reports worsening breathing over last 4 hours”, etc.	ED provider note; Admitting team admission note; Nephrology consult note; Cardiology consult note **[must be within 24h of adm]**	
**Presenting O_2 sat <92%**					
**Instructions**			List saturation value (even if above 92%), Ex. 91%, 100%	ED provider note; Admitting team admission note; Nephrology consult note; Cardiology consult note **[must be within 24h of adm; select first available value]**	

Physical Exam/ Clinical Findings of Volume Overload

Finding	Y	N	Qualitative	Date and Time	Chart location/ source
**Bibasilar lung rales**					
**Instructions**			List description	ED provider note; Admitting team admission note; Nephrology consult note; Cardiology consult note **[must be within 24h of adm]**	
**3rd heart sound**					
**Instructions**			List description	ED provider note; Admitting team admission note; Nephrology consult note; Cardiology consult note **[must be within 24h of adm]**	
**Jugular vein distension**					
**Instructions**			List description	ED provider note; Admitting team admission note; Nephrology consult note; Cardiology consult note **[must be within 24h of adm]**	

MD Diagnosis	Y	N	Qualitative	Date and Time	Chart location/ source
**Diagnosis of heart failure or fluid overload in impression and plan**					
**Instructions**			List qualitative description and where mentioned in note- listed in impression, in plan, etc.	ED provider note; Admitting team admission note; Nephrology consult note; Cardiology consult note **[must be within 24h of adm]**	

Imaging Findings of Volume Overload

Finding	Y	N	Qualitative	Date and Time	Chart location/ source
**Interstitial edema**					**Indication:**
**Instructions**			If XR- note if portable; Fluid-related impression, ex: "worsening perihilar opacities which may reflect worsening edema"	Chest imaging: CXR or CT; Abdominal imaging: AXR or CT **[must be within 24h of adm]**	
**Bilateral infiltrate without fever**					**Indication:**
**Instructions**			If XR- note if portable; Fluid-related impression if not listed above; List temperature value (38.2 or 100.4)	Chest imaging: CXR or CT; Abdominal imaging: AXR or CT; Temperature in ED provider note; Admitting team admission note: Nephrology consult note; Cardiology consult note **[must be within 24h of adm; select 1st available value of temp]**	
**Pleural effusion**					**Indication:**
**Instructions**			If XR- note if portable; List size, descriptors, Ex: "unchanged blunting of bilat costophrenic angles, stable bilat pl effusions"	Chest imaging: CXR or CT; Abdominal imaging: AXR or CT **[must be within 24h of adm; select 1st available value]**	
**Abdominal ascites**					**Indication:**
**Instructions**			If XR- note if portable List amount/ size	Abdominal imaging: AXR or CT; **NOT an exam finding of ascites **[must be within 24h of adm]**	
**CVP >12 mmHg**					
**Instructions**			List value	Transducer from a central venous catheter- ED provider note; Admitting team admission note; Nephrology consult note; Cardiology consult note **[must be within 24h of adm]**	
**PCWP >12 mmHg**					
**Instructions**			List value	Swan-Ganz catheter- ED provider note; Admitting team admission note; Nephrology consult note; Cardiology consult note **[must be within 24h of adm]**	

**If multiple imaging types per patient within first 24 hours, review all and record data from exam with most detailed pulmonary imaging/ reporting

Echocardiograms

	Y	N	Indication/ type	EF	IVC	CVP	Effusion	Change	Date and Time
**TTE/ TEE this admission**									
**Most recent prior TTE/ TEE in system**									
**Instructions**			List indication if given; TTE vs. TEE	(%)	Dilated? Phasic respiratory change?	Estimated CVP (mmHg)	Pericardial effusion (yes/ no); size	Change from prior TTE/ TEE?- any noted change but look for change in EF or change in valve function or presence of vegetation (specify change)	Does **NOT** have to be within 24h of admission. If >1, use first available
