# Supplementary material for: Identification of volume overload hospitalizations among hemodialysis patients using administrative claims: a validation study
Source: BMC Nephrol. 2016 Nov 11;17:173. doi: 10.1186/s12882-016-0384-6 (PMC5105303; doi:10.1186/s12882-016-0384-6)
Supplement: Additional file 2: — Supplemental tables and figures. Description of data: Includes the following supplemental tables and figures: Table S1. Detailed description of claims-based definitions for volume-related hospitalizations used in prior studies. Table S2. Administrative claims-based definitions for volume overload hospital admissions constructed with ICD-9 discharge diagnosis codes and a dialysis procedure codes. Table S3. Validity of administrative claims definitions for volume overload hospital admissions with an additional dialysis procedure requirement. Table S4. Validity of administrative claims definitions for volume overload hospital admissions among hemodialysis patients admitted on or after October 1, 2010. Table S5. Validity of administrative claims definitions for volume overload hospital admissions among hemodialysis patients with Medicare insurance. Figure S1. Prevalence of volume overload admissions identified by administrative claims definitions with the additional dialysis procedure requirement. (PDF 462 kb) [file 12882_2016_384_MOESM2_ESM.pdf]

<u>**SUPPLEMENTAL MATERIAL**</u>

Table S1. Detailed description of claims-based definitions for volume-related hospitalizations used in prior studies

Previous study reference	Claims-based definition(s) for volume-related hospital admissions
**Banerjee D *et al*. Clin J Am Soc Nephrol. 2007 Nov;2(6):1186-90**	**Volume overload hospital admissions were defined as:**<br>• An inpatient admission with an ICD-9 discharge diagnosis of *fluid overload (276.6)*<br>• ICD-9 discharge diagnosis code position was not specified, but is likely any position because the terminology used to described events in the abstract was “hospitalization with”<br>**Note:** *Heart failure (428.x) and pulmonary edema (518.4)* hospitalizations, defined in a similar manner, were also assessed as outcomes in this study
**Arneson TJ *et al*. Clin J Am Soc Nephrol. 2010 Jun;5(6):1054-63**	**Volume overload treatment episodes were defined as:**<br>• A claim for an episode of care in an inpatient, hospital observation or emergency department setting with a primary discharge diagnosis of *fluid overload (276.6), heart failure (428, 402.x1, 404.x1, 404.x3, 398.91)* <u>or</u> *pulmonary edema (518.4, 514) with dialysis performed on the day of admission or the following day (specific dialysis procedure codes utilized to identify inpatient dialysis sessions were not documented)*<br>• Additional exclusion criteria that were applied:<br>− In primary analyses, hospital-based encounters with length of stay of >5 days were excluded (length of stay thresholds of >4 days and >3 days were evaluated in sensitivity analyses)<br>− Hospital-based encounters where the need for emergent fluid overload was precipitated by an acute cardiac event, as indicated by cardiovascular procedures or acute myocardial infarction, vascular access problems or any medical problem requiring surgical treatment were excluded (the extensive lists of the corresponding ICD-9 diagnosis and procedure codes, CPT procedure codes, and surgical DRG codes to define these conditions/procedures are listed in Appendix 3 of the Arneson et al.)
**Weinhandl *et al*. Am J Kidney Dis. 2015 Jun;65(6 Suppl 1):Svi, S1-140**	**Volume overload hospital admissions were defined in 2 ways:**<br>• Inpatient hospital admission with an ICD-9 discharge diagnosis code of *fluid overload (276.6, 276.69)* <u>or</u> *pleural effusion (511.9* present in the primary billing position only<br>• Inpatient hospital admission with an ICD-9 discharge diagnosis code of *fluid overload (276.6, 276.69)* <u>or</u> *pleural effusion (511.9)* present in the primary or leading secondary billing position

<u>Abbreviations:</u> CPT, Current Procedural Terminology; DRG, Diagnosis Related Group; ICD-9, International Classification of Diseases, Ninth Revision

Table S2. Administrative claims-based definitions for volume overload hospital admissions constructed with ICD-9 discharge diagnosis codes and a dialysis procedure codes

Definition number and description[a]	ICD-9 discharge diagnosis or CPT procedure codes[b]
**1D.** Fluid overload[c] with dialysis on the day of admission or the following day	276.6, 276.69 with 90935, 90937, 90945 and 90947 billed on the day of admission or the following day
**2D.** Pulmonary edema with dialysis on the day of admission or the following day	514, 518.4 with 90935, 90937, 90945 and 90947 billed on the day of admission or the following day
**3D.** Heart failure with dialysis on the day of admission or the following day	398.91, 402.x1, 404.x1, 404.x3, 428[d] with 90935, 90937, 90945 and 90947 billed on the day of admission or the following day
**4D.** Fluid overload[c] or pulmonary edema with dialysis on the day of admission or the following day	276.6, 276.69 or 514, 518.4 with 90935, 90937, 90945 and 90947 billed on the day of admission or the following day
**5D.** Fluid overload[c] or pleural effusion with dialysis on the day of admission or the following day	276.6, 276.69 or 511.9 with 90935, 90937, 90945 and 90947 billed on the day of admission or the following day
**6D.** Fluid overload[c] or heart failure with dialysis on the day of admission or the following day	276.6, 276.69 or 398.91, 398.91, 402.x1, 404.x1, 404.x3, 428[d] with 90935, 90937, 90945 and 90947 billed on the day of admission or the following day
**7D.** Fluid overload[c], pulmonary edema or heart failure with dialysis on the day of admission or the following day	276.6, 276.69 or 514, 518.4 or 398.91, 402.x1, 404.x1, 404.x3, 428[d] with 90935, 90937, 90945 and 90947 billed on the day of admission or the following day

[a] Definition numbers with a 'D' indicate that the claims-based definition had an additional criteria of a billed dialysis CPT procedure code for dialysis on the day of admission or the following day.

[b] Separate analyses evaluating definition validity considered ICD-9 diagnosis codes in: 1) *any billing order position*, 2) *the primary position only* and 3) *the primary and leading secondary positions*.

[c] Prior to October 1, 2010 the ICD-9 discharge diagnosis code 276.6 (fluid overload) was the only applicable code in existence. On October 1, 2010, ICD-9 diagnosis code 276.6 (fluid overload) became invalid and was replaced by more granular codes: 276.61 (transfusion associated circulatory overload) and 276.69 (other fluid overload). For hospitalizations with a discharge date prior to October 1, 2010 the ICD-9 code 276.6 was used to construct claims-based volume overload definitions. For hospitalizations with a discharge date on or after October 1, 2010 the ICD-9 code 276.69 was used to construct claims-based volume overload definitions.

[d] Specified three digit ICD-9 diagnosis categories included all existing 4th and 5th digit diagnosis codes.

Abbreviations: CPT, Current Procedural Terminology; ICD-9, International Classification of Diseases, Ninth Revision

Table S3. Validity of administrative claims definitions for volume overload hospital admissions with an additional dialysis procedure requirement

Claims-based definition	n (%)[a]	SENS (95% CI)[b]	SPEC (95% CI)[b]	PPV (95% CI)[b]	NPV (95% CI)[b]
**ICD-9 discharge codes could be *in any position***					
**1.** Fluid overload	29 (9.4)	32.0 (21.7, 43.8)	97.9 (95.1, 99.3)	82.8 (64.2, 94.2)	81.8 (76.8, 86.1)
**1D.** Fluid overload with dialysis on the day of admission or the following day[c]	19 (6.1)	21.3 (12.7, 32.3)	98.7 (96.3, 99.7)	84.2 (60.4, 96.6)	79.7 (74.6, 84.1)
**2.** Pulmonary edema	12 (3.9)	10.7 (4.7, 19.9)	98.3 (95.7, 99.5)	66.7 (34.9, 90.1)	77.4 (72.3, 82.1)
**2D.** Pulmonary edema with dialysis on the day of admission or the following day[c]	10 (3.2)	9.3 (3.8, 18.3)	98.7 (96.3, 99.7)	70.0 (34.8, 93.3)	77.3 (72.1, 81.9)
**3.** Heart failure	87 (28.2)	53.3 (41.4, 64.9)	79.9 (74.2, 84.9)	46.0 (35.2, 57.0)	84.2 (78.8, 88.8)
**3D.** Heart failure with dialysis on the day of admission or the following day[c]	41 (13.3)	28.0 (18.2, 39.6)	91.5 (87.1, 94.7)	51.2 (35.1, 67.1)	79.9 (74.5, 84.5)
**4.** Fluid overload or pulmonary edema	38 (12.3)	40.0 (28.9, 52.0)	96.6 (93.4, 98.5)	78.9 (62.7, 90.4)	83.4 (78.4, 87.6)
**4D.** Fluid overload or pulmonary edema with dialysis on the day of admission or the following day[c]	26 (8.4)	28.0 (18.2, 39.6)	97.9 (95.1, 99.3)	80.8 (60.6, 93.4)	80.9 (75.8, 85.3)
**5.** Fluid overload or pleural effusion	38 (12.3)	41.3 (30.1, 53.3)	97.0 (93.9, 98.8)	81.6 (65.7, 92.3)	83.8 (78.8, 87.9)
**5D.** Fluid overload or pleural effusion with dialysis on the day of admission or the following day[c]	25 (8.1)	28.0 (18.2, 39.6)	98.3 (95.7, 99.5)	84.0 (63.9, 95.5)	81.0 (75.9, 85.4)
**6.** Fluid overload or heart failure	110 (35.6)	77.3 (66.2, 86.2)	77.8 (71.9, 82.9)	52.7 (43.0, 62.3)	91.5 (86.7, 94.9)
**6D.** Fluid overload or heart failure with dialysis on the day of admission or the following day[c]	56 (18.1)	44.0 (32.5, 55.9)	90.2 (85.6, 93.7)	58.9 (45.0, 71.9)	83.4 (78.2, 87.8)
**7.** Fluid overload, pulmonary edema or heart failure	116 (37.5)	82.7 (72.2, 90.4)	76.9 (71.0, 82.2)	53.4 (44.0, 62.8)	93.3 (88.8, 96.4)
**7D.** Fluid overload, pulmonary edema or heart failure with dialysis on the day of admission or the following day[c]	61 (19.7)	49.3 (37.6, 61.1)	89.7 (85.1, 93.3)	60.7 (47.3, 72.9)	84.7 (79.6, 88.9)
**ICD-9 discharge codes could be *in primary position only***					
**1.** Fluid overload	5 (1.6)	6.7 (2.2, 14.9)	100.0 (98.4, 100.0)	100.0 (47.8, 100.0)	77.0 (71.8, 81.6)
**1D.** Fluid overload with dialysis on the day of admission or the following day[c]	4 (1.3)	5.3 (1.5, 13.1)	100.0 (98.4, 100.0)	100.0 (39.8, 100.0)	76.7 (71.6, 81.3)
**2.** Pulmonary edema	4 (1.3)	5.3 (1.5, 13.1)	100.0 (98.4, 100.0)	100.0 (39.8, 100.0)	76.7 (71.6, 81.3)

**2D.** Pulmonary edema <u>with dialysis</u> on the day of admission or the following day[c]	4 (1.3)	5.3 (1.5, 13.1)	100.0 (98.4, 100.0)	100.0 (39.8, 100.0)	76.7 (71.6, 81.3)
**3.** Heart failure	10 (3.2)	12.0 (5.6, 21.6)	99.6 (97.6, 100.0)	90.0 (55.5, 99.7)	77.9 (72.8, 82.5)
**3D.** Heart failure <u>with dialysis</u> on the day of admission or the following day[c]	7 (2.3)	8.0 (3.0, 16.6)	99.6 (97.6, 100.0)	85.7 (42.1, 99.6)	77.2 (72.0, 81.8)
**4.** Fluid overload <u>or</u> pulmonary edema	9 (2.9)	12.0 (5.6, 21.6)	100.0 (98.4, 100.0)	100.0 (66.4, 100.0)	78.0 (72.9, 82.6)
**4D.** Fluid overload <u>or</u> pulmonary edema <u>with dialysis</u> on the day of admission or the following day[c]	8 (2.6)	10.7 (4.7, 19.9)	100.0 (98.4, 100.0)	100.0 (63.1, 100.0)	77.7 (72.6, 82.3)
**5.** Fluid overload <u>or</u> pleural effusion	6 (1.9)	6.7 (2.2, 14.9)	99.6 (97.6, 100.0)	83.3 (35.9, 99.6)	76.9 (71.7, 81.5)
**5D.** Fluid overload <u>or</u> pleural effusion <u>with dialysis</u> on the day of admission or the following day[c]	5 (1.6)	5.3 (1.5, 13.1)	99.6 (97.6, 100.0)	80.0 (28.4, 99.5)	76.6 (71.5, 81.3)
**6.** Fluid overload <u>or</u> heart failure	15 (4.9)	18.7 (10.6, 29.3)	99.6 (97.6, 100.0)	93.3 (68.1, 99.8)	79.3 (74.2, 83.7)
**6D.** Fluid overload <u>or</u> heart failure <u>with dialysis</u> on the day of admission or the following day[c]	11 (3.6)	13.3 (6.6, 23.2)	99.6 (97.6, 100.0)	90.9 (58.7, 99.8)	78.2 (73.1, 82.7)
**7.** Fluid overload, pulmonary edema <u>or</u> heart failure	19 (6.1)	24.0 (14.9, 35.3)	99.6 (97.6, 100.0)	94.7 (74.0, 99.9)	80.3 (75.3, 84.8)
**7D.** Fluid overload, pulmonary edema <u>or</u> heart failure <u>with dialysis</u> on the day of admission or the following day[c]	15 (4.9)	18.7 (10.6, 29.3)	99.6 (97.6, 100.0)	93.3 (68.1, 99.8)	79.3 (74.2, 83.7)
**ICD-9 discharge codes could be *<u>in primary or leading secondary positions</u>***					
**1.** Fluid overload	5 (1.6)	6.7 (2.2, 14.9)	100.0 (98.4, 100.0)	100.0 (47.8, 100.0)	77.0 (71.8, 81.6)
**1D.** Fluid overload <u>with dialysis</u> on the day of admission or the following day[c]	4 (1.3)	5.3 (1.5, 13.1)	100.0 (98.4, 100.0)	100.0 (39.8, 100.0)	76.7 (71.6, 81.3)
**2.** Pulmonary edema	6 (1.9)	8.0 (3.0, 16.6)	100.0 (98.4, 100.0)	100.0 (54.1, 100.0)	77.2 (72.1, 81.8)
**2D.** Pulmonary edema <u>with dialysis</u> on the day of admission or the following day[c]	6 (1.9)	8.0 (3.0, 16.6)	100.0 (98.4, 100.0)	100.0 (54.1, 100.0)	77.2 (72.1, 81.8)
**3.** Heart failure	15 (4.9)	17.3 (9.6, 27.8)	99.1 (96.9, 99.9)	86.7 (59.5, 98.3)	78.9 (73.8, 83.4)
**3D.** Heart failure <u>with dialysis</u> on the day of admission or the following day[c]	8 (2.6)	9.3 (3.8, 18.3)	99.6 (97.6, 100.0)	87.5 (47.3, 99.7)	77.4 (72.2, 82.0)
**4.** Fluid overload <u>or</u> pulmonary edema	11 (3.6)	14.7 (7.6, 24.7)	100.0 (98.4, 100.0)	100.0 (71.5, 100.0)	78.5 (73.4, 83.0)
**4D.** Fluid overload <u>or</u> pulmonary edema <u>with dialysis</u> on the day of admission or the following day[c]	10 (3.2)	13.3 (6.6, 23.1)	100.0 (98.4, 100.0)	100.0 (69.2, 100.0)	78.3 (73.1, 82.8)

**5.** Fluid overload or pleural effusion	6 (1.9)	6.7 (2.2, 14.9)	99.6 (97.6, 100.0)	83.3 (35.9, 99.6)	76.9 (71.7, 81.5)
**5D.** Fluid overload or pleural effusion with dialysis on the day of admission or the following day[c]	5 (1.6)	5.3 (1.5, 13.1)	99.6 (97.6, 100.0)	80.0 (28.4, 99.5)	76.6 (71.5, 81.3)
**6.** Fluid overload or heart failure	20 (6.5)	24.0 (14.9, 35.3)	99.1 (96.9, 99.9)	90.0 (68.3, 98.8)	80.3 (75.2, 84.7)
**6D.** Fluid overload or heart failure with dialysis on the day of admission or the following day[c]	12 (3.9)	14.7 (7.6, 24.7)	99.6 (97.6, 100.0)	91.7 (61.5, 99.8)	78.5 (73.3, 83.0)
**7.** Fluid overload, pulmonary edema or heart failure	26 (8.4)	32.0 (21.7, 43.8)	99.1 (96.9, 99.9)	92.3 (74.9, 99.1)	82.0 (77.0, 86.3)
**7D.** Fluid overload, pulmonary edema or heart failure with dialysis on the day of admission or the following day[c]	18 (5.8)	22.7 (13.8, 33.8)	99.6 (97.6, 100.0)	94.4 (72.7, 99.9)	80.1 (75.0, 84.5)

[a] Prevalence of volume overload admissions identified by each respective administrative claims definition in the study cohort. Definition numbers with a ‘D’ indicate that the claims-based definition had an additional criteria of a billed dialysis CPT procedure code for dialysis on the day of admission or the following day.

[b] Validity estimates and 95% CIs are expressed as percentages. Clinically adjudicated volume overload events, as outlined in Figure 1, served as the reference standard. Patients admitted and discharged on the same day were excluded from this analysis because we were unable to determine if they received dialysis on the day following admission using inpatient administrative claims data. In this subgroup of 309 patients, there were 75 adjudicated volume overload admissions.

[c] Definition numbers with a ‘D’ indicate that the claims-based definition had an additional criteria of a billed dialysis CPT procedure code for dialysis on the day of admission or the following day for the identification of volume overload admissions and are defined in Supplemental Table 1.

Abbreviations: 95% CI, 95% confidence interval; CPT, Current Procedural Terminology; ICD-9, International Classification of Diseases, Ninth Revision; NPV, negative predictive value; PPV, positive predictive value; SENS, sensitivity; SPEC, specificity.

Table S4. Validity of administrative claims definitions for volume overload hospital admissions among hemodialysis patients admitted on or after October 1, 2010

Claims-based definition	n (%)[a]	SENS (95% CI)[b]	SPEC (95% CI)[b]	PPV (95% CI)[b]	NPV (95% CI)[b]
**ICD-9 discharge codes could be *in any position***					
**1.** Fluid overload	25 (8.7)	30.3 (19.6, 42.9)	97.7 (94.8, 99.2)	80.0 (59.3, 93.2)	82.4 (77.3, 86.8)
**2.** Pulmonary edema	12 (4.2)	12.1 (5.4, 22.5)	98.2 (95.4, 99.5)	66.7 (34.9, 90.1)	78.9 (73.6, 83.6)
**3.** Heart failure	76 (26.5)	53.0 (40.3, 65.4)	81.4 (75.7, 86.3)	46.1 (34.5, 57.9)	85.3 (79.8, 89.8)
**4.** Fluid overload or pulmonary edema	33 (11.5)	37.9 (26.2, 50.7)	96.4 (93.0, 98.4)	75.8 (57.7, 88.9)	83.9 (78.7, 88.1)
**5.** Fluid overload or pleural effusion	34 (11.8)	40.9 (29.0, 53.7)	96.8 (93.6, 98.7)	79.4 (62.1, 91.3)	84.6 (79.5, 88.8)
**6.** Fluid overload or heart failure	95 (33.1)	74.2 (62.0, 84.2)	79.2 (73.2, 84.3)	51.6 (41.1, 62.0)	91.1 (86.2, 94.8)
**7.** Fluid overload, pulmonary edema or heart failure	101 (35.2)	80.3 (68.7, 89.1)	78.3 (72.3, 83.5)	52.5 (42.3, 62.5)	93.0 (88.3, 96.2)
**ICD-9 discharge codes could be *in primary position only***					
**1.** Fluid overload	4 (1.4)	6.1 (1.7, 14.8)	100.0 (98.3, 100.0)	100.0 (39.8, 100.0)	78.1 (72.8, 82.8)
**2.** Pulmonary edema	5 (1.7)	7.6 (2.5, 16.8)	100.0 (98.3, 100.0)	100.0 (47.8, 100.0)	78.4 (73.1, 83.0)
**3.** Heart failure	9 (3.1)	12.1 (5.4, 22.5)	99.5 (97.5, 100.0)	88.9 (51.8, 99.7)	79.1 (73.9, 83.8)
**4.** Fluid overload or pulmonary edema	9 (3.1)	13.6 (6.4, 24.3)	100.0 (98.3, 100.0)	100.0 (66.4, 100.0)	79.5 (74.3, 84.1)
**5.** Fluid overload or pleural effusion	5 (1.7)	6.1 (1.7, 14.8)	99.5 (97.5, 100.0)	80.0 (28.4, 99.5)	78.0 (72.7, 82.7)
**6.** Fluid overload or heart failure	13 (4.5)	18.2 (9.8, 29.6)	99.5 (97.5, 100.0)	92.3 (64.0, 99.8)	80.3 (75.1, 84.8)
**7.** Fluid overload, pulmonary edema or heart failure	18 (6.3)	25.8 (15.8, 38.0)	99.5 (97.5, 100.0)	94.4 (72.7, 99.9)	81.8 (76.6, 86.2)
**ICD-9 discharge codes could be *in primary position only***					
**1.** Fluid overload	4 (1.4)	6.1 (1.7, 14.8)	100.0 (98.3, 100.0)	100.0 (39.8, 100.0)	78.1 (72.8, 82.8)
**2.** Pulmonary edema	7 (2.4)	10.6 (4.4, 20.6)	100.0 (98.3, 100.0)	100.0 (59.0, 100.0)	78.9 (73.7, 83.6)
**3.** Heart failure	13 (4.5)	16.7 (8.6, 27.9)	99.1 (96.8, 99.9)	84.6 (54.6, 98.1)	79.9 (74.7, 84.5)
**4.** Fluid overload or pulmonary edema	11 (3.8)	16.7 (8.6, 27.9)	100.0 (98.3, 100.0)	100.0 (71.5, 100.0)	80.1 (74.9, 84.6)
**5.** Fluid overload or pleural effusion	5 (1.7)	6.1 (1.7, 14.8)	99.5 (97.5, 100.0)	80.0 (28.4, 99.5)	78.0 (72.7, 82.7)
**6.** Fluid overload or heart failure	17 (5.9)	22.7 (13.3, 34.7)	99.1 (96.8, 99.9)	88.2 (63.6, 98.5)	81.1 (75.9, 85.6)
**7.** Fluid overload, pulmonary edema or heart failure	24 (8.4)	33.3 (22.2, 46.0)	99.1 (96.8, 99.9)	91.7 (73.0, 99.0)	83.3 (78.2, 87.6)

[a] Prevalence of volume overload admissions identified by each respective administrative claims definition among sampled patients admitted on or after October 1, 2010 (n = 287).

[b] Validity estimates and 95% CIs are expressed as percentages. Clinically adjudicated volume overload events, as outlined in Figure 1, served as the reference standard. In this subgroup of 287 patients, there were 66 adjudicated volume overload admissions.

Abbreviations: 95% CI, 95% confidence interval; ICD-9, International Classification of Diseases, Ninth Revision; NPV, negative predictive value; PPV, positive predictive value; SENS, sensitivity; SPEC, specificity.

Supplemental Table 5. Validity of administrative claims definitions for volume overload hospital admissions among hemodialysis patients with Medicare insurance

Claims-based definitions	n (%)[a]	SENS (95% CI)[b]	SPEC (95% CI)[b]	PPV (95% CI)[b]	NPV (95% CI)[b]
**ICD-9 discharge codes could be *in any position***					
**1.** Fluid overload	25 (9.4)	32.3 (20.9, 45.3)	97.5 (94.4, 99.1)	80.0 (59.3, 93.2)	82.6 (77.2, 87.1)
**2.** Pulmonary edema	8 (3.0)	6.5 (1.8,15.7)	98.0 (95.1, 99.5)	50.0 (15.7, 84.3)	77.5 (71.9, 82.4)
**3.** Heart failure	77 (28.9)	56.5 (43.3, 69.0)	79.4 (73.2, 84.7)	45.5 (34.1, 57.2)	85.7 (79.9, 90.4)
**4.** Fluid overload or pulmonary edema	30 (11.3)	35.5 (23.7, 48.7)	96.1 (92.4, 98.3)	73.3 (54.1, 87.7)	83.1 (77.6, 87.6)
**5.** Fluid overload or pleural effusion	34 (12.8)	43.5 (31.0, 56.7)	96.6 (93.1, 98.6)	79.4 (62.1, 91.3)	84.9 (79.6, 89.3)
**6.** Fluid overload or heart failure	97 (36.5)	80.6 (68.6, 89.6)	77.0 (70.6, 82.6)	51.5 (41.2, 61.8)	92.9 (87.9, 96.3)
**7.** Fluid overload, pulmonary edema or heart failure	100 (37.6)	82.2 (70.5, 90.8)	76.0 (69.5, 81.7)	51.0 (40.8, 61.1)	93.4 (88.5, 96.6)
**ICD-9 discharge codes could be *in primary position only***					
**1.** Fluid overload	4 (1.5)	6.5 (1.8, 15.7)	100.0 (98.2, 100.0)	100.0 (39.8, 100.0)	77.9 (72.3, 82.7)
**2.** Pulmonary edema	2 (0.8)	3.2 (0.4, 11.2)	100.0 (98.2, 100.0)	100.0 (15.8, 100.0)	77.3 (71.7, 82.2)
**3.** Heart failure	10 (3.8)	14.5 (6.9, 25.8)	99.5 (97.3, 100.0)	90.0 (55.5, 99.7)	79.3 (73.8, 84.1)
**4.** Fluid overload or pulmonary edema	6 (2.3)	9.7 (3.6, 19.9)	100.0 (98.2, 100.0)	100.0 (54.1, 100.0)	78.5 (73.0, 83.3)
**5.** Fluid overload or pleural effusion	5 (1.9)	6.5 (1.8, 15.7)	99.5 (97.3, 100.0)	80.0 (28.4, 99.5)	77.8 (72.2, 82.7)
**6.** Fluid overload or heart failure	14 (5.3)	21.0 (11.7, 33.2)	99.5 (97.3, 100.0)	92.9 (66.1, 99.8)	80.6 (75.1, 85.3)
**7.** Fluid overload, pulmonary edema or heart failure	16 (6.0)	24.2 (14.2, 36.7)	99.5 (97.3, 100.0)	93.8 (69.8, 99.8)	81.2 (75.8, 85.8)
**ICD-9 discharge codes could be *in primary position only***					
**1.** Fluid overload	4 (1.5)	6.5 (1.8, 15.7)	100.0 (98.2, 100.0)	100.0 (39.8, 100.0)	77.9 (72.3, 82.7)
**2.** Pulmonary edema	3 (1.1)	4.8 (1.0, 13.5)	100.0 (98.2, 100.0)	100.0 (29.2, 100.0)	77.6 (72.0, 82.5)
**3.** Heart failure	15 (5.6)	21.0 (11.7, 33.2)	99.0 (96.5, 99.9)	86.7 (59.5, 98.3)	80.5 (75.0, 85.2)
**4.** Fluid overload or pulmonary edema	7 (2.6)	11.3 (4.7, 21.9)	100.0 (98.2, 100.0)	100.0 (59.0, 100.0)	78.8 (73.3, 83.6)
**5.** Fluid overload or pleural effusion	5 (1.9)	6.5 (1.8, 15.7)	99.5 (97.3, 100.0)	80.0 (28.4, 99.5)	77.8 (72.2, 82.7)
**6.** Fluid overload or heart failure	19 (7.1)	27.4 (16.9, 40.2)	99.0 (96.5, 99.9)	89.5 (66.9, 98.7)	81.8 (76.4, 86.4)
**7.** Fluid overload, pulmonary edema or heart failure	22 (8.3)	32.3 (20.9, 45.3)	99.0 (96.5, 99.9)	90.9 (70.8, 98.9)	82.8 (77.5, 87.3)

[a] Prevalence of volume overload admissions identified by each respective administrative claims definition among sampled patients with Medicare as their primary payer (n = 266).

[b] Validity estimates and 95% CIs are expressed as percentages. Clinically adjudicated volume overload events, as outlined in Figure 1, served as the reference standard. In this subgroup of 266 patients, there were 62 adjudicated volume overload admissions.

Abbreviations: 95% CI, 95% confidence interval; ICD-9, International Classification of Diseases, Ninth Revision; NPV, negative predictive value; PPV, positive predictive value; SENS, sensitivity; SPEC, specificity

Figure S1. Prevalence of volume overload admissions identified by administrative claims definitions with the additional dialysis procedure requirement

A. ICD-9 CODES IN ANY POSITION

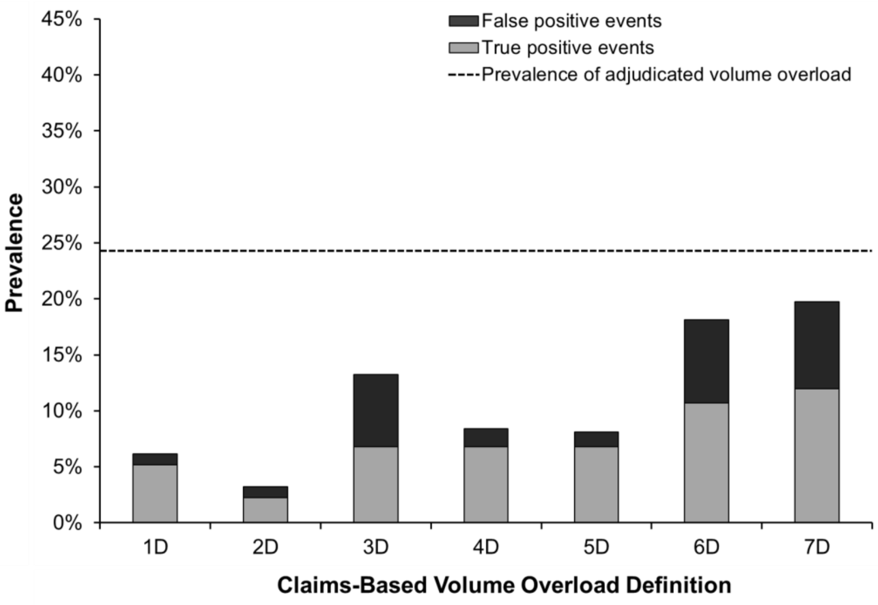

B. ICD-9 CODES IN THE PRIMARY POSITION

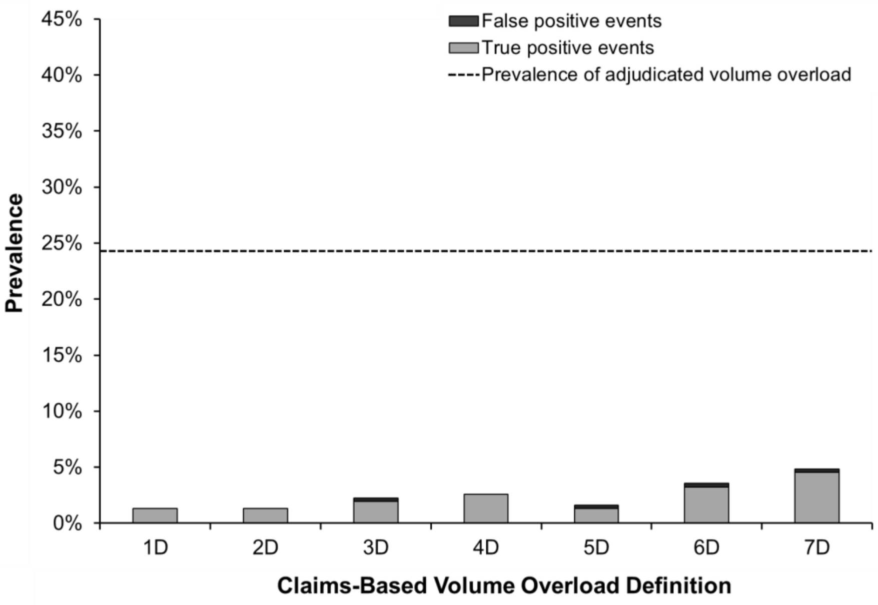

C. ICD-9 CODES IN THE PRIMARY OR LEADING SECONDARY POSITION

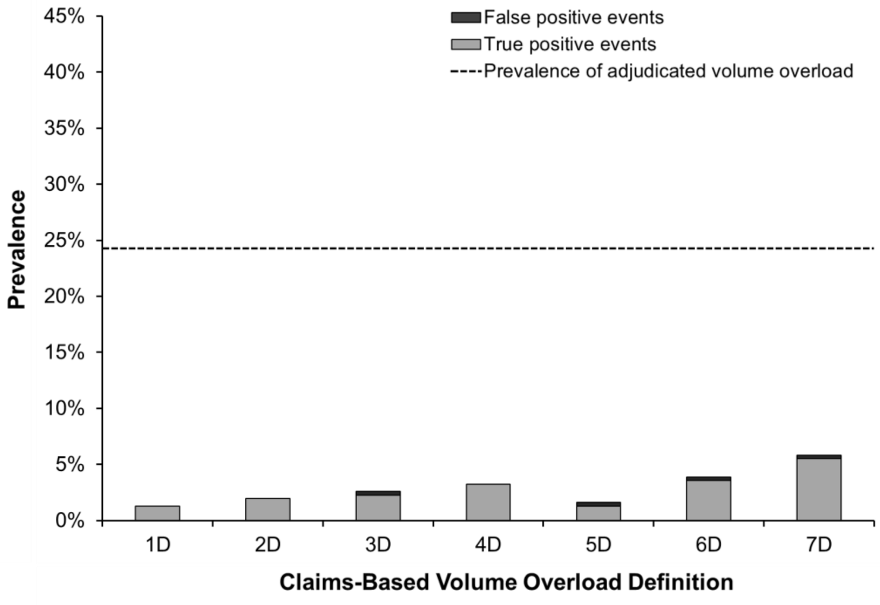

The claims-based volume overload definition numbers on the x-axis correspond to the following definitions: 1D) fluid overload with dialysis on the day of admission or the following day; 2D) pulmonary edema with dialysis on the day of admission or the following day; 3D) heart failure with dialysis on the day of admission or the following day; 4D) fluid overload or pulmonary edema with dialysis on the day of admission or the following day; 5D) fluid overload or pleural effusion with dialysis on the day of admission or the following day, 6D) fluid overload or heart failure with dialysis on the day of admission or the following day; and 7D) fluid overload, pulmonary edema or heart failure with dialysis on the day of admission or the following day. Prevalence was computed among patients admitted from January 1, 2010 and June 30, 2013 with hospital length of stay ≥1 day (n = 309). Patients admitted and discharged on the same day were excluded from this analysis because we were unable to determine if they received dialysis on the day following admission using inpatient administrative claims data. The total bar height (light gray portion + dark gray portion) represents the prevalence of volume overload admissions in the study cohort identified using the specified claims-based definitions. Panel A depicts prevalence estimates when claims-based definitions were constructed considering ICD-9 discharge diagnosis codes in any position. Panel B depicts prevalence estimates when claims-based definitions were constructed considering discharge diagnosis codes in the primary position only. Panel C depicts prevalence estimates when claims-based definitions were constructed considering discharge diagnosis codes in the primary or leading secondary position.
